# Low-Cost Optical–Inertial Point Cloud Acquisition and Sketch System

**DOI:** 10.3390/s26020476

**Published:** 2026-01-11

**Authors:** Tung-Chen Chao, Hsi-Fu Shih, Chuen-Lin Tien, Han-Yen Tu

**Affiliations:** 1 Department of Mechanical Engineering, National Chung Hsing University, Taichung 40227, Taiwan; kenny3271879@gmail.com; 2 Department of Electrical Engineering, Feng Chia University, Taichung 40724, Taiwan; cltien@fcu.edu.tw; 3 Department of Electrical Engineering, Chinese Culture University, Taipei 11114, Taiwan

**Keywords:** three-dimensional (3D) measurement, point cloud, optical displacement sensor, inertial sensor, accelerometer, gyroscope, attitude angle, Mahoney complementary filtering

## Abstract

This paper proposes an optical three-dimensional (3D) point cloud acquisition and sketching system, which is not limited by the measurement size, unlike traditional 3D object measurement techniques. The system employs an optical displacement sensor for surface displacement scanning and a six-axis inertial sensor (accelerometer and gyroscope) for spatial attitude perception. A microprocessor control unit (MCU) is responsible for acquiring, merging, and calculating data from the sensors, converting it into 3D point clouds. Butterworth filtering and Mahoney complementary filtering are used for sensor signal preprocessing and calculation, respectively. Furthermore, a human–machine interface is designed to visualize the point cloud and display the scanning path and measurement trajectory in real time. Compared to existing works in the literature, this system has a simpler hardware architecture, more efficient algorithms, and better operation, inspection, and observation features. The experimental results show that the maximum measurement error on 2D planes is 4.7% with a root mean square (RMS) error of 2.1%, corresponding to the reference length of 10.3 cm. For 3D objects, the maximum measurement error is 5.3% with the RMS error of 2.4%, corresponding to the reference length of 9.3 cm. Finally, it was verified that this system can also be applied to large-sized 3D objects for outlines.

## 1. Introduction

Length measurement is a very basic but important technology. The traditional way is to use a ruler for measurement. If it is a hard ruler, it can only measure straight-line lengths on flat surfaces. If it is a soft ruler, such as a tape measure, it can measure non-straight lengths, but because the ruler needs to fit closely with the object to be measured, whether the two are closely matched will affect the accuracy of the measurement results. With the progress of measuring technology, the contact measurement using a ruler is gradually replaced by non-contact optical measurement. For example, a laser distance measuring device can directly illuminate the target with a beam to calculate the distance of a straight line. However, because the beam is moving forward in a straight line, this type of measuring device can usually only measure the length of a straight line and cannot measure that of any non-straight or non-flat objects.

**Figure 1:**
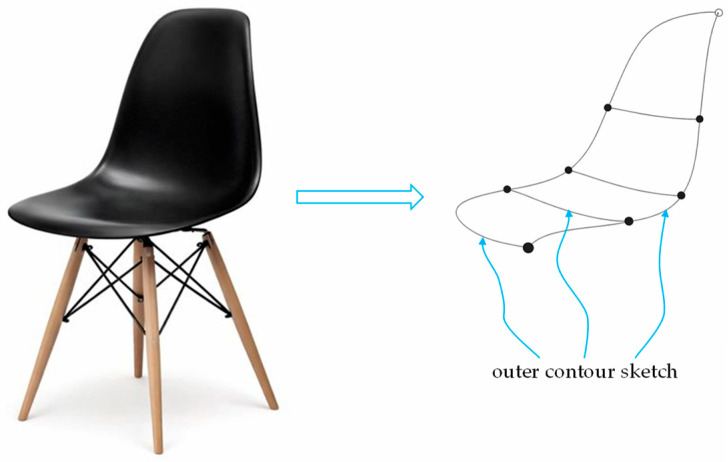
Sampling diagram of the outer contour of a solid object, where the right image shows the contour drawn from the left chair.

The measurement of non-straight or non-flat objects is often an unavoidable requirement in engineering. Especially for arbitrarily shaped three-dimensional (3D) objects, it is not easy to measure any trajectory on its surface, let alone the spatial stereoscopic information implicit in the trajectory. Figure 1 shows a common reverse engineering product development diagram. The left picture is a solid object, and the right picture is the pattern drawn by appearance sampling for outer contour sketch. For example: construction and civil engineers need to measure irregular boundaries of architectural designs or cadastral maps, industrial design engineers need to measure the dimensions of objects with curved surfaces for product reverse development, clothing design engineers and packaging industry need to draw patterns for fabric or cover material cutting of irregular shapes, interior design engineers need to saw or carve decorative wood material, etc. Most of them currently use a tape-measure to make rough measurements, and there are no convenient and accurate 3D curve measurement tools. Although reverse engineering can use an optical 3D scanner to scan an object from multiple different angles to obtain the 3D outline, and then capture the trajectory from the generated point cloud data. The equipment includes: the time-of-flight (TOF) method [1,2], the structured light method [3,4], and the stereo vision method [5,6]. These methods are mainly used for 3D scanning of the entire objects. If they are only used for curve, trajectory, or contour capturing, the process seems complicated and impractical. Moreover, the equipment is expensive, the structure is complex, and the operation and processing procedures are cumbersome. It also has strict limitations on the external dimensions of the object being measured. It cannot be easily adopted by all walks of life. Therefore, the development of dedicated measurement tools is more pragmatic and necessary.

In order to achieve the purpose of non-straight length measurement, M. Barbaric et al. developed a contact measurement method [7,8], using a roller mechanism designed at one end of a pen-type device to connect a counter, and calculating the corresponding number of turns by the roller around the object to be measured. The length of the moving trajectory can be obtained by multiplying the number of rotations of the roller by the circumference of the roller. It has the characteristic of measuring curve or trajectory lengths of arbitrary 3D objects but the roller needs to be in good contact with the surface of the object, so the surface characteristics greatly affect the measurement results and there are several shortcomings existing. The measurement values obtained perhaps not represent the true path length of the object to be measured. In addition, this measurement method cannot provide the spatial stereoscopic information of the trajectory. Bagel-Labs company developed a digital ruler. It combines a tape measure, a roller, and a sonar to provide measurement of any curved or irregular surface object with a tape ruler up to 3 m of measuring range, a scroll wheel up to 10 m of measuring range, or even a sonar sensor to measure out-of-reach targets [9]. Looking at the above two creations, although the length of a curve or a curved surface can be measured, the common feature is that the measurement is made by contacting the surface, which cannot avoid the shortcomings mentioned above. Therefore, in order to solve these problems, using non-contact measurement methods, especially using optical architecture with spatial orientation sensors, should be the most feasible method. In addition, the above-mentioned creations can only calculate the linear length value, and cannot provide the spatial stereoscopic information of the trajectory change at each position, such as coordinate values (*x*, *y*, *z*), rotation amount (yaw, pitch, roll), etc., the measurement assistance for reverse engineering is quite limited. Under the consideration, length measurement that can generate point cloud data is a feasible method. Using point cloud data, not only can the non-straight length be calculated, but its stereoscopic shape and spatial information can also be obtained.

D. Grivon et al. proposed a low cost OptInertial 3D scanner by combining an optical sensor with three inertial units (accelerometer, magnetometer, and gyroscope) [10]. It can measure the 3D shape of the object and record its movement by a control unit for obtaining point cloud. They tested a low-curvature surface of 640 mm × 160 mm and achieved a maximum error of 4–5 mm. Due to the physical constraints from surfaces to be measured, the probe size of the hardware structure needs to be further improved. B. Milosevic et al. used a combination of stereo cameras and three inertial sensors to obtain line segments of curves and combine them into 3D sketches [11]. The stereo cameras were combined with binocular depth measurement technology to calculate the pen tip coordinates and direction of the measurement equipment. Also, the sensing device is equipped with four infrared light emitters to help the device position during image recognition. The system is complex and expensive. The measured objects are limited by the field of view and depth of field of the stereo camera and the illumination of the light source. T. Stanko et al. combined an inertial sensing system with an incremental wheel to obtain the coordinates of the point cloud in 3D space, and further fitted it into a mesh surface structure [12]. Inertial sensing includes a three-axis accelerometer and a three-axis magnetometer, which are used to sense the normal vector of the surface to be measured, and capture the normal vector data once after the incremental wheel rolls a preset fixed distance on the surface to be measured. At the same time, it is necessary to define through numbers whether the scroll is currently located on the edge or inside the object, which is used to facilitate data classification during post-processing.

In today’s human–machine interaction and intelligent systems, real-time processing of acquired 3D point cloud data plays a crucial role, especially in assisting hand function rehabilitation and highlighting the importance of 3D point cloud data in dynamic and complex scenes [13,14]. In addition to the aforementioned articles, there are several other papers related to spatial sensing technology, which will not be discussed in detail here [15,16,17,18,19,20,21,22].

Based on the above literature review, it can be seen that using optical and inertial sensors to obtain point cloud data for generating the shape or contour trajectory of an object is a reliable and effective method, and is worthy of further exploration in this research. Compared to existing literature, this study proposes a simpler hardware architecture that generates point clouds using only a single six-axis inertial sensor combined with an optical displacement sensor. Furthermore, this study employs more efficient filters and algorithms and integrates a PI feedback controller to reduce computational complexity and improve performance. Simultaneously, this study also develops a human–machine stereoscopic display interface and real-time 3D point cloud and trajectory display functions to facilitate user inspection and observation.

## 2. System Design and Methods

**Figure 2:**
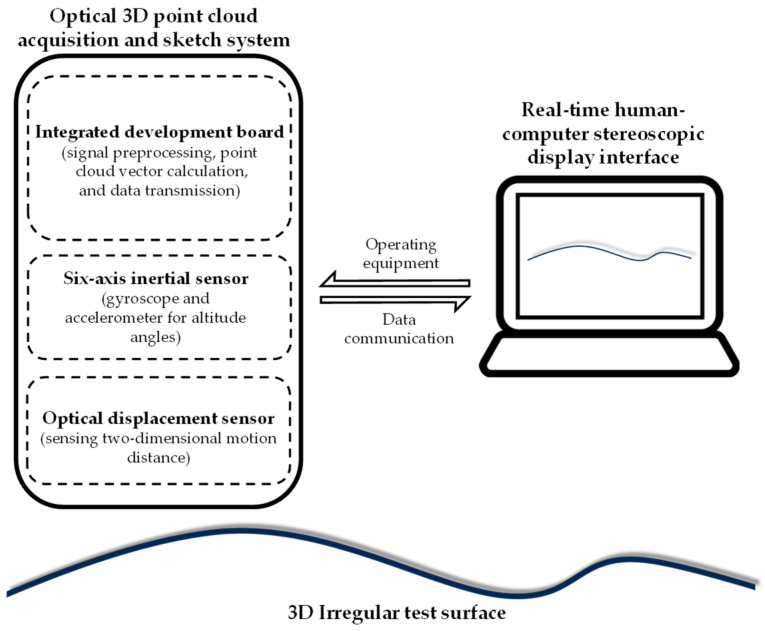
Configuration of the proposed optical 3D point cloud acquisition and sketch system.

Configuring a 3D point cloud acquisition system for shape or contour generation of objects with arbitrary surfaces is the purpose of the study. Figure 2 shows the design concept. The system includes an optical displacement sensor and a six-axis inertial spatial sensor. Regarding optical displacement sensors, optical sensing modules used for motion detection inside optical mice are mature, readily available, and cost-effective components, and many documents have explored the use of this component for various sensing applications [23,24,25,26,27,28,29,30,31,32,33,34,35,36]. It can be used for providing distance changes in motion in this study. As for inertial space sensors, they mainly consist of g-sensors, gyroscopes, magnetometers, and e-compasses. Early versions of these sensors were inconvenient due to their large size, but recent advancements in microelectromechanical systems (MEMS) design and manufacturing technologies have led to their miniaturization and widespread application across various fields [37,38].

### 2.1. System Design

**Figure 3:**
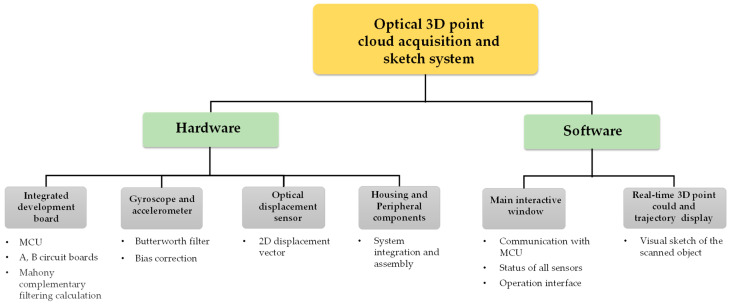
System architecture of the proposed optical 3D point cloud acquisition and sketch system.

Figure 3 illustrates the overall system architecture of this study, comprising two main parts: hardware and software design. In the hardware design, the system employs an optical displacement sensor for surface displacement scanning and an inertial sensor for spatial attitude sensing. A microprocessor control unit (MCU) captures signals from the sensing elements, merges and calculates the data, and converts it into 3D point cloud coordinate data. In the software design, a real-time human–machine interface is designed to visualize the point cloud coordinate data captured and calculated by the hardware, display the scanning path and measurement trajectory on a computer screen in real time, and allow the acquisition of selected measurement data.

### 2.2. Attitude Angle Calculation

Attitude angles are the angular changes of coordinates between a measurement system and a reference system when describing an object [39]. Two coordinate systems are needed, one representing the object’s space and the other the fixed space: the body-fixed coordinate system (object space) and the navigation coordinate system (fixed space). When an object deflects in space, the sensor data output from the inertial sensor relative to the body-fixed coordinate system can be converted into a coordinate rotation matrix after calculating the attitude angle. This transforms the planar vector from the displacement sensor into a 3D spatial vector relative to the navigation coordinate system, thus obtaining point cloud coordinate values.

**Figure 4:**
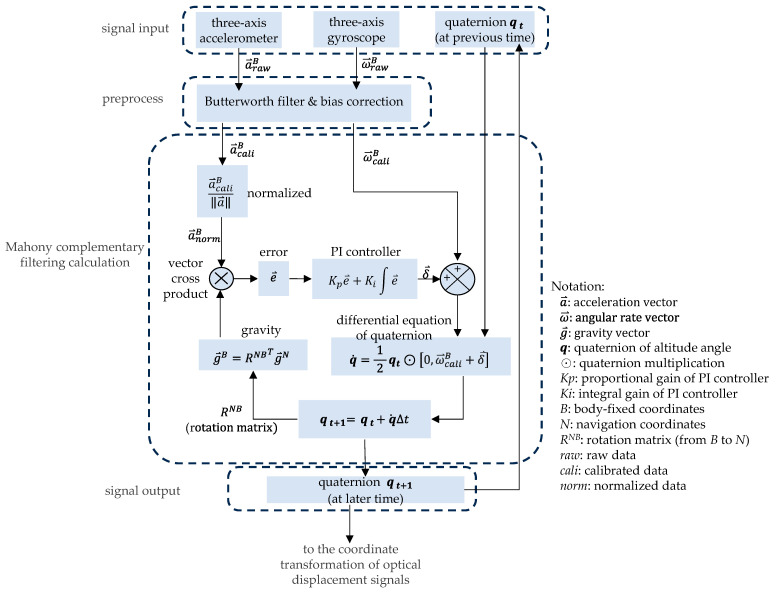
Calculation method for generating altitude angles.

Common methods for obtaining attitude angles include Euler angles, rotation matrices, and quaternion operations; relevant theories can be found in related literature [40,41,42,43]. This study uses a six-axis inertial sensor (GY-512, MPU-6050) (which internally contains a three-axis accelerometer unit and a three-axis gyroscope unit) and an optical displacement sensor (ADNC-9800) for optical mouse applications. Figure 4 illustrates the calculation method for converting the output signals of the two sensor units into attitude angles. In the figure, the three-axis acceleration signals *α* and angular rate signals *ω* output by the accelerometer and gyroscope, respectively, are first processed by a Butterworth filter and a bias correction preprocessing procedure before entering the Mahony complementary filtering calculation process. The acceleration signals are first normalized and then cross-produced with the gravity vector, which is transformed and rotated from the final quaternion attitude angle ***q*** to obtain the error between these two vectors. The error is then fed back to the gyroscope’s angular rate signals by the proportional–integral (PI) controller. The corrected gyroscope angular rate signals are converted into a quaternion vector and multiplied by the previous quaternion attitude angle vector to calculate the differential quaternion value of the attitude angle, and then integrated to obtain the instantaneous quaternion attitude angle vector at later time. This quaternion attitude angle vector can be directly provided to the optical displacement sensor for coordinate transformation calculations, and the corresponding coordinate rotation matrix can also be calculated from its components, as Equation (1).

(1) $$R^{NB}=\left[\begin{array}{ccc} 1-2(q_{y}^{2}+q_{z}^{2}) & 2(q_{x}q_{y}-q_{w}q_{z}) & 2(q_{x}q_{z}+q_{w}q_{y})\\ 2(q_{x}q_{y}+q_{w}q_{z}) & 1-2(q_{x}^{2}+q_{z}^{2}) & 2(q_{y}q_{z}{-q}_{w}q_{x})\\ 2(q_{x}q_{z}-q_{w}q_{y}) & 2(q_{y}q_{z}+q_{w}q_{x}) & 1-2(q_{x}^{2}+q_{y}^{2}) \end{array}\right]$$

where *R^NB^* is the rotation matrix from the body-fixed coordinates to the navigation coordinates and [*q_w_*, *q_x_*, *q_y_*, *q_z_*] is the quaternion attitude angle vector ***q_t_*_+1_** of the body-fixed coordinates related to the navigation coordinates. The parameters related to attitude angle calculation are shown in Table 1.

### 2.3. 3D Coordinate Reconstruction

**Figure 5:**
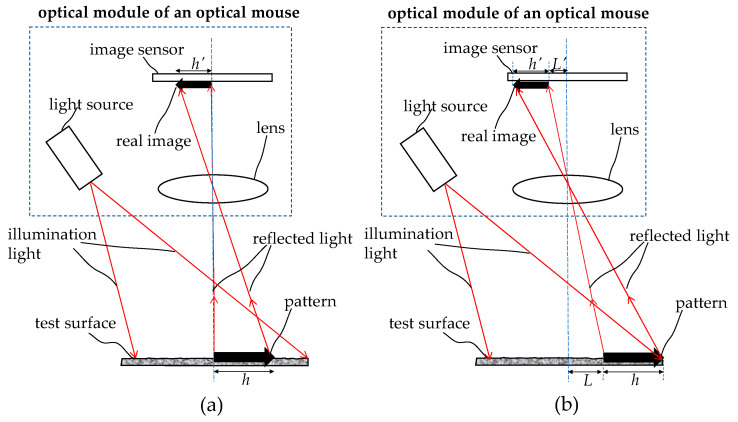
Principle of displacement sensing using the optical module. (**a**) the object–image (bold arrows) relationship before movement, (**b**) the object–image (bold arrows) relationship after movement.

Figure 5 illustrates the principle of displacement sensing using the optical module of an optical mouse. Figure 5a shows the initial position of the sensor on the test object. The light source illuminates a pattern of length *h* on the test surface, and the reflected light is reflected by the optical lens to produce a real image *h’* on the image sensing element. The magnification of this imaging system is *M*. Since the image sensing element is composed of a two-dimensional (2D) pixel array, and the length and width of each pixel are known fixed values, the length *h’* of the real image detected by the image sensing element can be used to calculate the length *h* of the pattern on the test surface through the magnification of the imaging system. Figure 5b illustrates the imaging relationship after the optical length measuring device moves a distance *L* to the left relative to the test surface from the starting position. Since the pattern moves a distance *L* to the right relative to the optical length measuring device, the real image moves relatively to the left by *L’* on the image sensing element within the optical length measuring device. The ratio of *L* to *L’* is still *M*. Therefore, through analysis and calculation by the image processing element, the actual moving distance *L* of the optical length measuring device can be derived.

**Figure 6:**
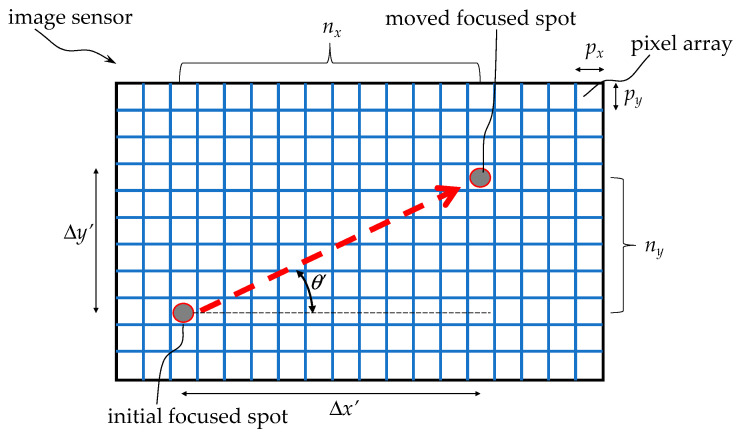
Relationship between the initial focused spot and the moved spot on the pixel array of the image sensor, where the red arrow indicates the moving path.

Figure 6 illustrates the pixel array on the image sensor, and the relationship between the initial focused spot and the moved focused spot of the real image converging on it. Each pixel in the pixel array has dimensions of *p_x_* and *p_y_*, respectively. The two focused spots are *n_x_* pixels apart on the *x*-axis (distance Δ*x*’) and *n_y_* pixels apart on the *y*-axis (distance Δ*y*’). The moving distance *L’* and the actual distance *L* on the image sensor can be calculated as the below Equations.

(2) $$L^{\prime }=[(∆x^{\prime }{)}^{2}+(∆y^{\prime }{)}^{2}{]}^{\frac{1}{2}}=[(n_{x}p_{x}{)}^{2}+(n_{y}p_{y}{)}^{2}{]}^{\frac{1}{2}}$$

(3) $$L=\frac{L^{\prime }}{M}$$

In addition to calculating the distance traveled, the direction angle *θ’* of the movement can also be determined from the distance traveled along the *x* and *y* axes.

(4) $$\theta^{\prime }=\arctan\left(\frac{∆y^{\prime }}{∆x^{\prime }}\right)=\arctan\left(\frac{n_{y}p_{y}}{n_{x}p_{x}}\right)$$

**Figure 7:**
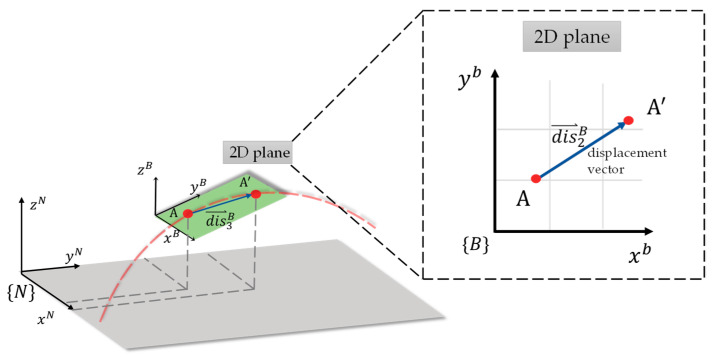
Transformation from 2D plane body-fixed coordinate displacement vector to 3D navigation coordinate displacement vector, where A represents the position before the move and A’ represents the position after the move.

Since optical displacement sensors can only capture 2D displacement vectors, attitude angle calculations are necessary to transform the 2D planar vectors in the body-fixed coordinate system into 3D spatial vectors in the navigation coordinate system. This approach differs slightly from common 3D coordinate reconstruction methods. Figure 7 illustrates the transformation, where {*B*} represents the body-fixed coordinate system and {*N*} represents the navigation coordinate system. Using the quaternion attitude angle vector obtained through Mahony complementary filtering as described above, a quaternion operation is performed using Equations (5)–(8) to obtain the transformed spatial displacement vector with the navigation coordinates as a reference, and point cloud and path data is acquired.

(5) $${\overset{⃑}{dis}}_{2}^{B}=[∆x,∆y]$$

(6) $${\overset{⃑}{dis}}_{3}^{B}=[∆x,∆y,\;0]$$

(7) $${dis}_{4}^{B}=[0,∆x,∆y,0]$$

where the 2D body-fixed coordinate displacement vector ${\overset{⃑}{dis}}_{2}^{B}$ is first converted into a 3D body-fixed coordinate displacement vector ${\overset{⃑}{dis}}_{3}^{B}$, then into a quaternion body-fixed coordinate displacement vector ${dis}_{4}^{B}$, and then multiplied with the quaternion attitude angle vector ***q_t_*_+1_** to convert it into a quaternion navigation coordinate displacement vector ${dis}_{4}^{N}$.

(8) $${dis}_{4}^{N}=q_{t+1}⊙{dis}_{4}^{B}{⊙(q_{t+1})}^{*}$$

where ⊙ denotes the quaternion multiplication. Since the quaternion attitude angle vector ***q_t_*_+1_** is a unit quaternion, no normalization operation is needed after the quaternion multiplication.

Alternatively, the aforementioned transformation matrix *R^NB^* can be used to directly convert the 3D body-fixed coordinate vector into the 3D navigation coordinate ${\overset{⃑}{dis}}_{3}^{N}$, as shown in Equation (9).

(9) $${\overset{⃑}{dis}}_{3}^{N}=R^{NB}{\overset{⃑}{dis}}_{3}^{B}$$

### 2.4. Error Propagation and Sensitivity Analysis

This study establishes a systematic quantitative error propagation model using variational Equations and perturbation theory to analyze the impact of attitude errors and displacement noise on spatial coordinate data. First, the discretized state recurrence Equation of the system is defined as Equation (10). $P_{t}^{N}$ and $P_{t-1}^{N}$ represents the point cloud coordinates at time *t* and previous time *t* − 1. *R^NB^*(*q_t_*) is rotation matrix from the body-fixed coordinates to the navigation coordinates at the quaternion attitude angle vector ***q_t_***. By introducing small perturbation terms into the point cloud coordinates, displacements, and rotation matrices, and neglecting higher-order terms based on the assumption of small errors, a linear error propagation of Equation (11) is derived, where *J_att_* and *J_dis_* are the Jacobian matrices for attitude and displacement, respectively, *δθ* is the small attitude estimation error, and $\delta {dis}_{3}^{B}$ is the small displacement error. The magnitudes of both Jacobian matrices can be seen as the sensitivities for attitude angles and displacement, respectively.

(10) $$P_{t}^{N}=P_{t-1}^{N}+R^{NB}(q_{t}){dis}_{3}^{B}$$

(11) $$\delta P_{t}^{N}=\delta P_{t-1}^{N}+J_{att}\delta \theta +J_{dis}\delta {dis}_{3}^{B}$$

During continuous measurement, the overall measurement error is the sum of the error terms of each sampled data point. According to the error propagation, the overall measurement error $\delta P_{Total}^{N}$ can be defined as the integral of the attitude sensitivity and displacement sensitivity with respect to the sensing error, as shown in Equation (12).

(12) $$\delta P_{Total}^{N}=\sum_{k=1}^{t}\left(J_{att,k}\delta \theta_{k}+J_{dis,k}{\delta dis}_{3,k}^{B}\right)$$

### 2.5. Hardware Architecture Design

**Figure 8:**
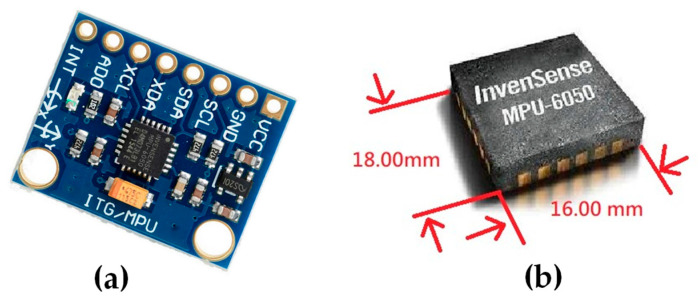
(**a**) GY-512 six-axis sensor containing an (**b**) MPU-6050 sensing chip.

**Figure 9:**
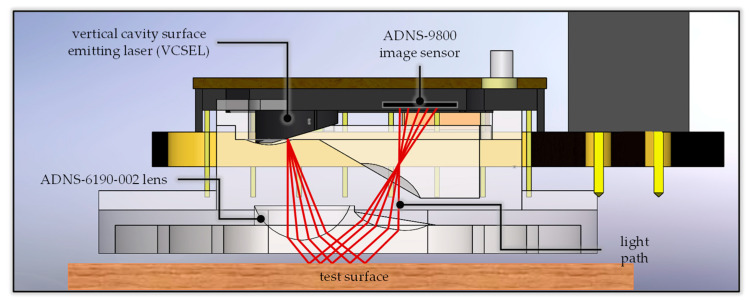
Optical displacement sensor composed of a light source, an ADNS-9800 chip, and an ADNS-6120-002 optical lens.

**Figure 10:**
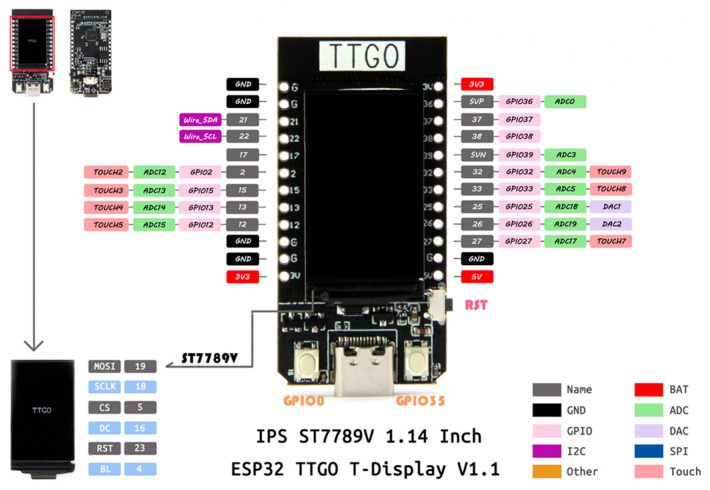
Integrated development board with the TTGO T-Display and ESP32 MCU.

**Figure 11:**
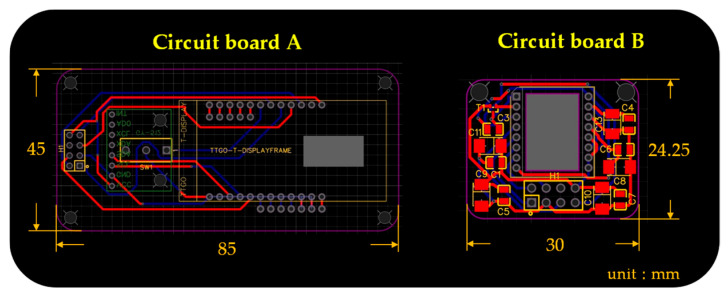
A and B circuit boards for the integrated development board, inertial sensor, and optical displacement sensor.

**Figure 12:**
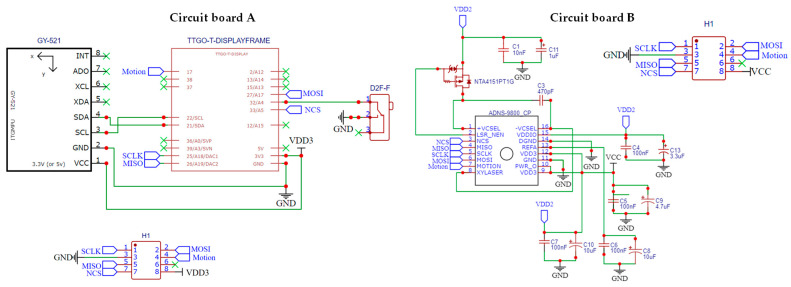
Circuit diagrams for the pin mapping of A and B boards.

**Figure 13:**
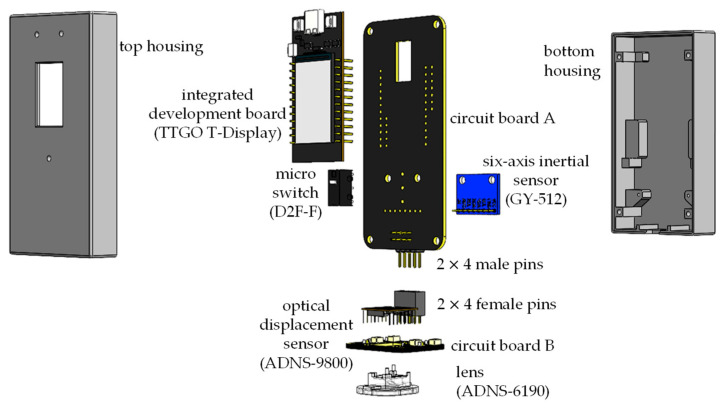
Overall hardware architecture design of the exploded view.

**Figure 14:**
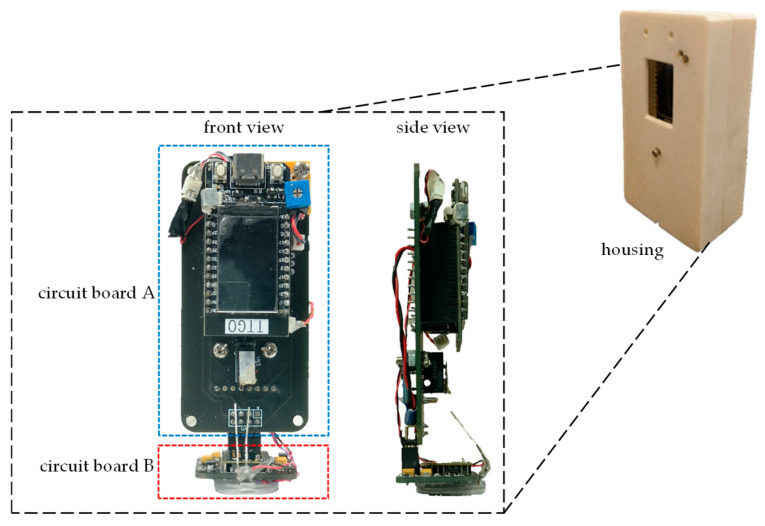
Completed physical entity of the proposed system.

**Figure 15:**
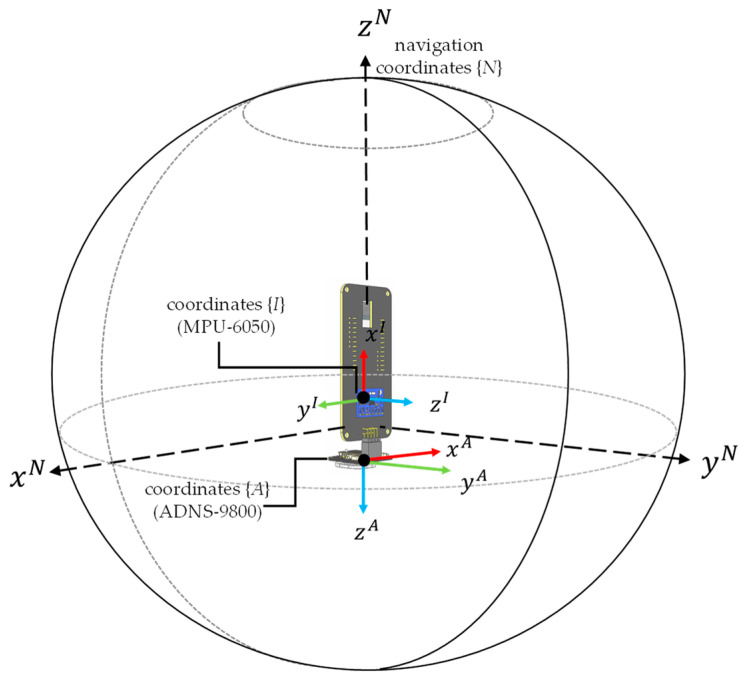
Relative relationships between the body-fixed coordinates and navigation coordinates of each sensing element.

The overall hardware design architecture of this study has been shown in Figure 3. A commercially available GY-512 sensor is selected, containing an MPU-6050 sensing chip manufactured by InvenSense Inc., providing sensing signals from a three-axis gyroscope and a three-axis accelerometer, as shown in Figure 8. An optical displacement sensor is composed of a light source and an ADNS-9800 chip [44], manufactured by PixArt Imaging Inc., paired with an ADNS-6190-002 optical lens [45], as shown in Figure 9. The MCU part uses the TTGO T-Display of integrated development board manufactured by Espressif Systems, containing an ESP32 and a TFT LCD display, as shown in Figure 10. The aforementioned components are integrated into two self-designed circuit boards, A and B, as shown in Figure 11. The circuit diagrams for the pin mapping of A and B boards are shown in Figure 12. Regarding data acquisition rate and communication protocol, the MPU-6050 transmits raw data via the 400 kHz I2C protocol, while the ADNS-9800 transmits data via the SPI protocol at 2 MHz. A housing was designed and fabricated using fused deposition modeling (FDM) 3D printing equipment. The overall hardware architecture design of the exploded view is shown in Figure 13, and the completed physical entity is shown in Figure 14. Figure 15 illustrates the relative relationships between the body-fixed coordinates and navigation coordinates of each sensing element within the physical architecture. The GY-512 sensor and the ADNS-9800 sensor are mounted on different circuit boards. The GY-512 sensor is fixed to board A, and the ADNS-9800 sensor is fixed to board B. They are secured at a 90-degree angle using right-angle brackets, and a level is used to check for any horizontal misalignment. The design ensures that the *x* and *y* coordinates of their centers overlap on the body-fixed coordinate system, with only the *z* coordinate having an offset. However, this offset does not affect the conversion of the 2D displacement vector from the body-fixed coordinate system to a 3D navigation coordinate system vector.

### 2.6. Real-Time Visual Human–Machine Stereoscopic Display Interface

**Figure 16:**
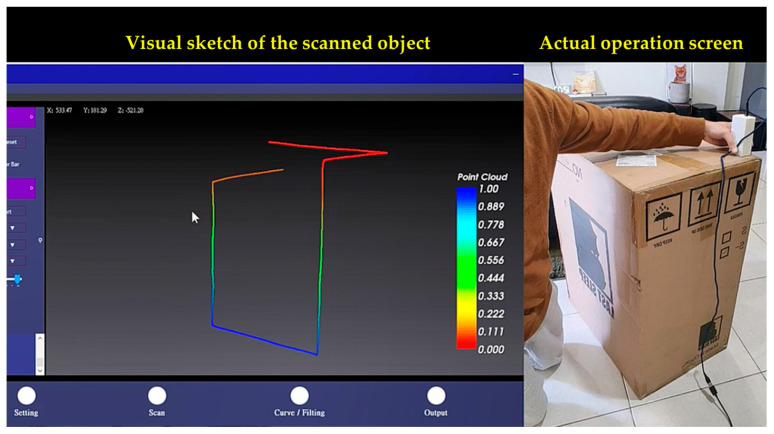
Completed window interface, where the left screen displays a visual sketch of the scanned object and the right screen displays the actual operation.

To visualize the spatial coordinates of the point cloud data acquired by the aforementioned sensing system and to plot the scanning trajectory and measurement path length, this study uses C# Windows Forms software to design a real-time human–machine stereoscopic display interface. Figure 16 shows the completed window interface, where the left screen displays a visual sketch of the scanned object and the right screen displays the actual operation.

## 3. System Analysis

### 3.1. Calibration Setup

**Figure 17:**
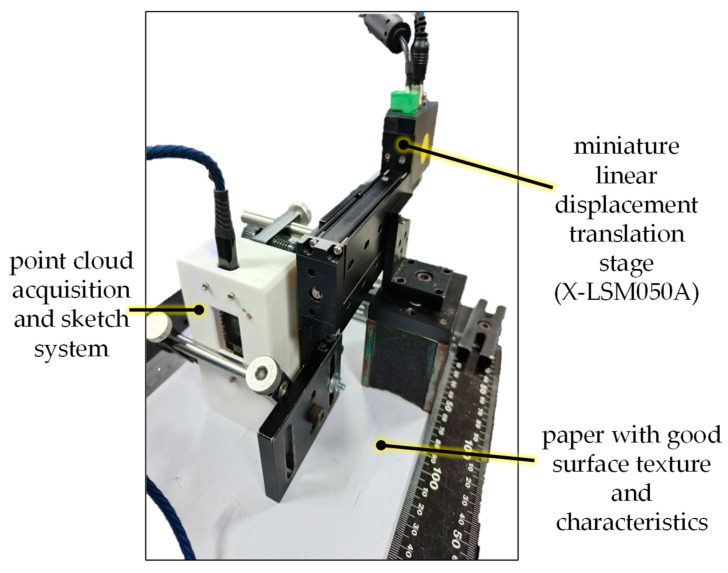
Setup for calibrating the completed point cloud acquisition and sketch system.

This calibration setup is used for measuring data on a 2D plane, and the results are analyzed and calibrated. First, a measurement environment for calibration and testing needs to be set up. The device used is a miniature linear displacement translation stage from Zaber, model X-LSM050A. As shown in Figure 17, the linear platform is fixed on a stable, flat plate, and the completed point cloud acquisition system is then firmly attached to the linear platform. This ensures that the displacement of the linear platform can effectively and stably drive the movement of the sensing device. Furthermore, the test surface uses paper with good surface texture and characteristics.

### 3.2. Optical Displacement Sensor Analysis

**Figure 18:**
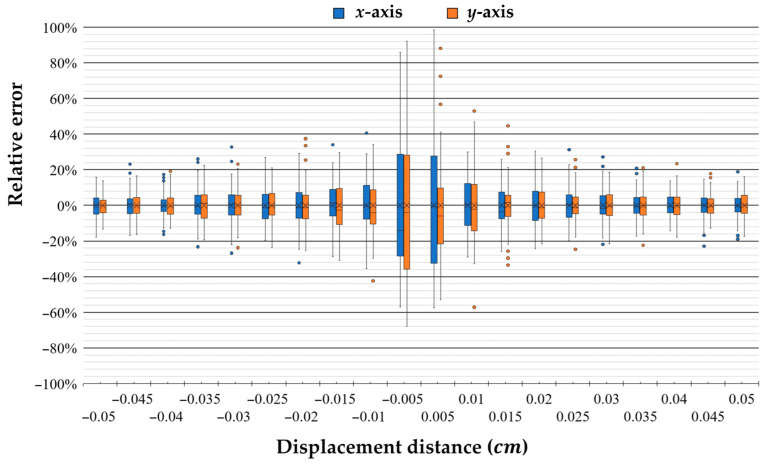
Short-distance measurement and data analysis on the *x* and *y* axes.

The ADNS-9800 chip can provide test surface images and surface quality (SQUAL) values (refer to the datasheet [44]). The SQUAL register is a measure of the number of valid features visible by the sensor in the current frame. By checking the SQUAL, adjusting the resistance of the illumination source to change the light intensity, and performing linear regression analysis, noise can be reduced and displacement correction can be achieved. Regarding firmware acceleration, the ADNS-9800 chip’s own shadow ROM (SROM) firmware can only use the officially released version. This study selected the SROM_A4 version with less displacement correction compensation and fixed the frame rate to obtain a relatively linear displacement data response. The completed point cloud acquisition system was used to acquire short-distance data on the *x* and *y* axes using the setup described in Figure 16 for verifying the resolution of the optical displacement sensor. The measurement data are shown in Figure 18. The relative error increased significantly when the displacement distance was less than 0.01 cm, and the reproducibility also diverged significantly. Therefore, this study selected 0.01 cm as the minimum resolution for sensing displacement changes.

According to the specifications of the optical lens module ADNS-6190-002 used in this study (refer to the datasheet [45]), only the working distance is listed, without optical parameters such as focal length. Therefore, it is difficult to calculate its magnification relationship. We can only ensure that the scanning process is at the optimal working distance position recommended in the specifications through mechanical design, and obtain the best and stable magnification relationship and working distance by using an optical displacement sensor correction procedure.

### 3.3. Inertial Sensor Analysis

**Figure 19:**
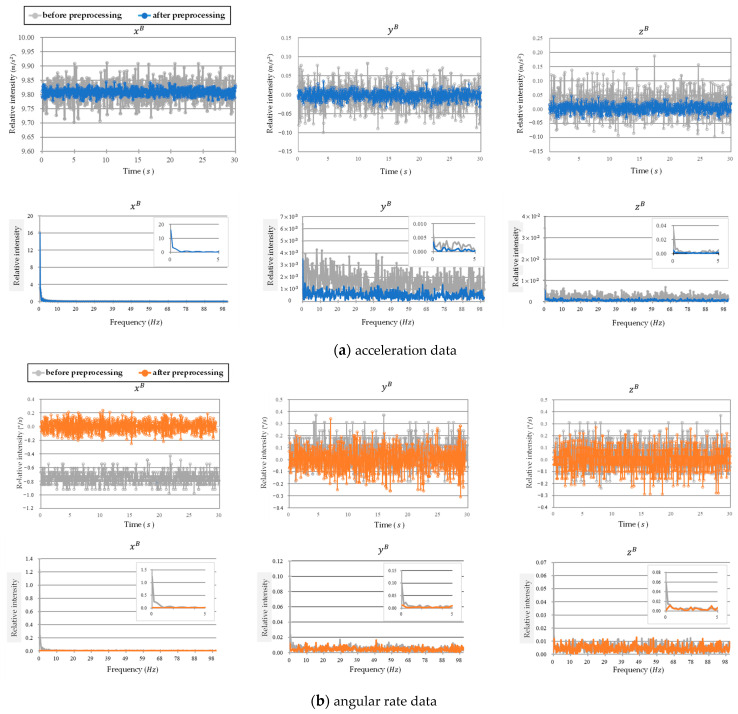
Comparison of (**a**) acceleration and (**b**) angular rate data before and after preprocessing.

The MPU-6050 has a full-scale angular velocity sensing range of ±250, ±500, ±1000, and ±2000°/sec (dps), enabling accurate tracking of both fast and slow motion. Its full-scale acceleration sensing range is ±2 g, ±4 g, ±8 g, and ±16 g. In this study, the raw data of acceleration and angular rate are first preprocessed using a bias compensation and low-pass Butterworth filter. The filtering results are shown in Figure 19. The noise of acceleration and angular rate is significantly filtered out, providing more stable and accurate data. As for the inertial sensor drift, the inertial sensor samples long-term continuous data under static conditions, analyzes its spectral distribution using fast Fourier transform (FFT), selects a cutoff frequency, and then feeds it into a high-pass Butterworth filter to filter out low-frequency inertial sensor drift errors. The first picture in Figure 19b shows an example of filtering drift for the angular rate of *x^B^*.

### 3.4. Parameter Settings of Sensors

Since the sensors used in this study are all standard products with complex specifications, readers can easily obtain their datasheets, so they will not be described separately in this article. Table 2 summarizes the settings of only a few important parameters used in the study.

### 3.5. Displacement Sensor Error Correction

**Figure 20:**
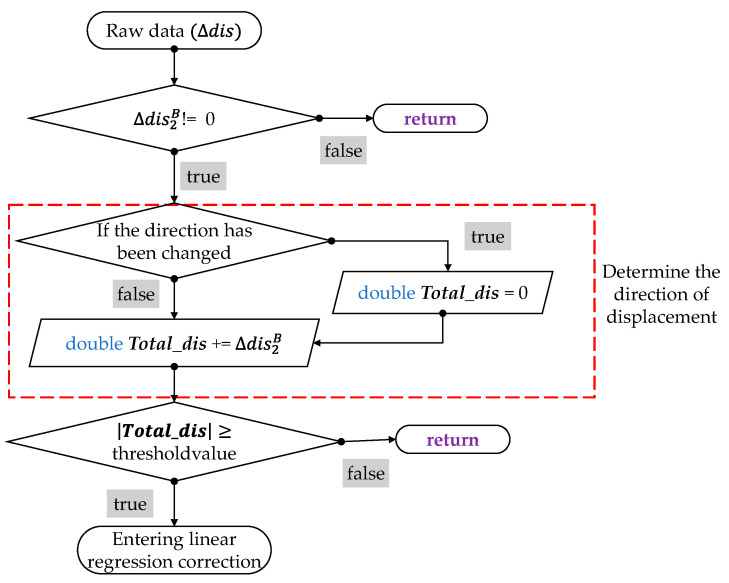
Flowchart of the threshold filter.

Since the sensed data is obtained by accumulating many tiny displacements, the correction of displacement sensing should start with small displacements and be adjusted gradually. First, a threshold filter is used to set the theoretical minimum resolution to increase stability. By measuring counts per inch (CPI) of displacement data converted to metric units, the arithmetic mean within three standard deviations for each axis is obtained as the threshold value, and this value is used for filtering as the flowchart in Figure 20. The threshold values are 0.0051 cm for the *x*-axis and 0.0047 cm for the *y*-axis, respectively.

Next, for the linear regression coefficients along different axes, the minute displacements of each threshold filter output were corrected to improve accuracy. The model used first-order linear regression; the intercept, as verified experimentally, showed that zero yielded better correction results. The slopes are shown in Table 3. The coefficient of determination (*R*^2^) was used as the criterion for selecting the regression Equation. The slope is the ratio of theoretical displacement to measured displacement; ideally, the slope *β*_1_ of the regression Equation should be close to ±1. After correction, the accuracy of the result was significantly improved.

Finally, the actual correction results were verified using a theoretical displacement of 5 cm as an example. The calibration effectiveness was evaluated by stability and accuracy: the former depends on the relative error between the data and the mean, while the latter is measured by the absolute error between the data and the theoretical displacement, as shown in Table 4.

### 3.6. Inertial Sensor Error Analysis

Balancing phase delay and computational complexity, a second-order filter (low-pass/high-pass filter) was selected as the signal processing model, and FFT analysis was performed on the raw acceleration and angular rate data. It was found that the main frequency components of hand movements were distributed between 9 and 16 Hz, while signal noise belonged to the high-frequency range. Therefore, 20 Hz was chosen as the cutoff frequency for the low-pass filter applied to acceleration. To suppress drift errors in angular velocity over long-term operation, optimal cutoff frequencies were defined for each axis as shown in Table 5. The threshold values were calculated by adding twice the standard deviation to the average values. Statistical analysis showed that data below the thresholds would cover 95% of the total data distribution, effectively identifying outliers.

## 4. Results

### 4.1. 2D Plane Measurement

**Figure 21:**
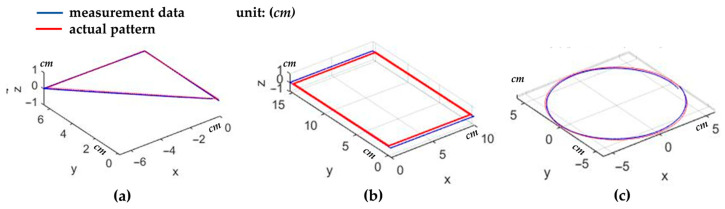
Comparison of the measurement data with the actual patterns for (**a**) right triangles, (**b**) rectangles, and (**c**) circles.

First, 2D planar measurements were performed using this system, measuring three patterns: right triangles, rectangles, and circles. Figure 21 shows a comparison of the measurement results with the actual patterns. The root mean square (RMS) errors of the calculated point cloud coordinates were 1.1% (0.053 cm), 2.1% (0.217 cm), and 2.8% (0.165 cm), respectively.

### 4.2. 3D Surface Measurement

**Figure 22:**
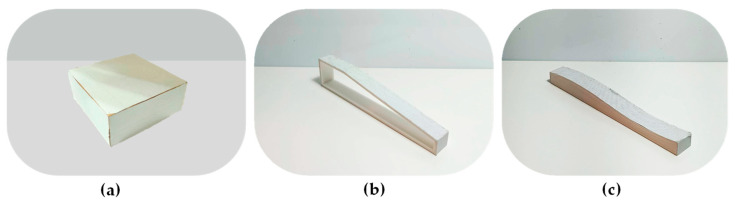
Photographs of the actual 3D-printed objects used for the measurements of (**a**) cuboids, (**b**) inclined surfaces, and (**c**) curved surfaces.

**Figure 23:**
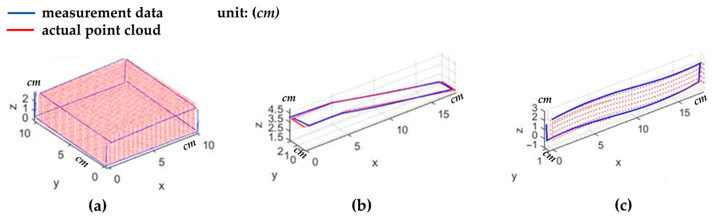
Comparison of the measurement data with the actual point cloud for (**a**) cuboids, (**b**) inclined surfaces, and (**c**) curved surfaces.

Next, 3D surface measurements were performed using this system, measuring three types of objects: cuboids, inclined surfaces, and curved surfaces. Figure 22 shows photographs of the actual 3D-printed objects used for the measurements. Figure 23 compares the measurement results with the dimensions of the actual objects. The RMS errors of the calculated point cloud coordinates were 2.3% (0.189 cm), 1.6% (0.171 cm), and 2.4% (0.221 cm), respectively.

### 4.3. Visual Point Cloud and Sketch Display

**Figure 24:**
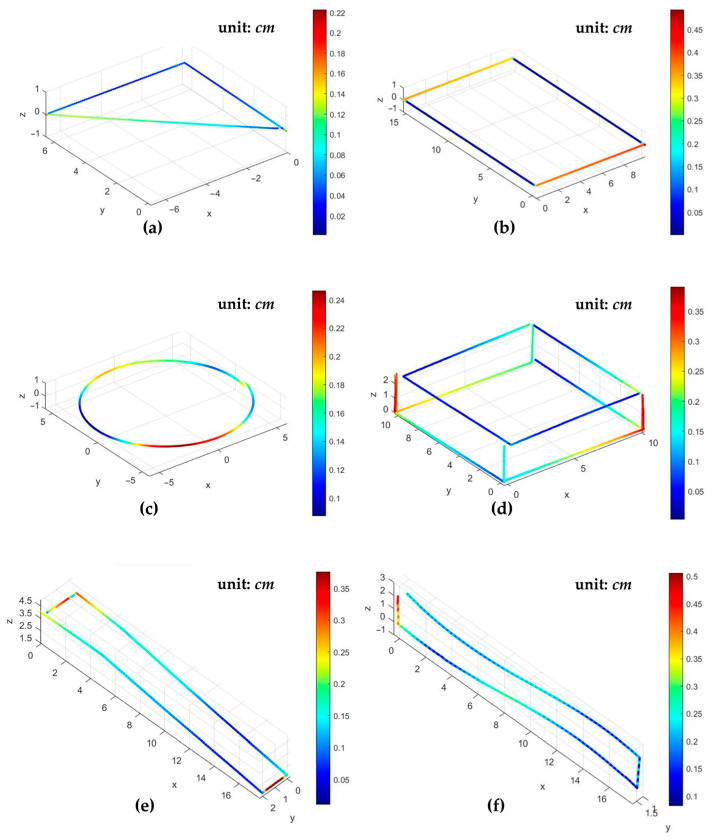
Point cloud and sketch images of the 2D and 3D measurements on the visual human–machine stereoscopic display interface, (**a**) a right triangle, (**b**) a rectangle, (**c**) a circle, (**d**) a cuboid, (**e**) an inclined surface, and (**f**) a curved surface.

Figure 24 shows the real-time point cloud and sketch images of the aforementioned 2D and 3D measurements on the visual human–machine stereoscopic display interface. Table 6 summarizes the measurement results. The table includes the total number of point clouds for each measurement object, the maximum error, and the RMS error of the measurement results. Furthermore, Table 7 compares the key features of the systems in the literature with the proposed system. The table shows that the distance resolution using an optical displacement sensor is significantly better than that without one.

### 4.4. Large Scale Object Measurement and Display

**Figure 25:**
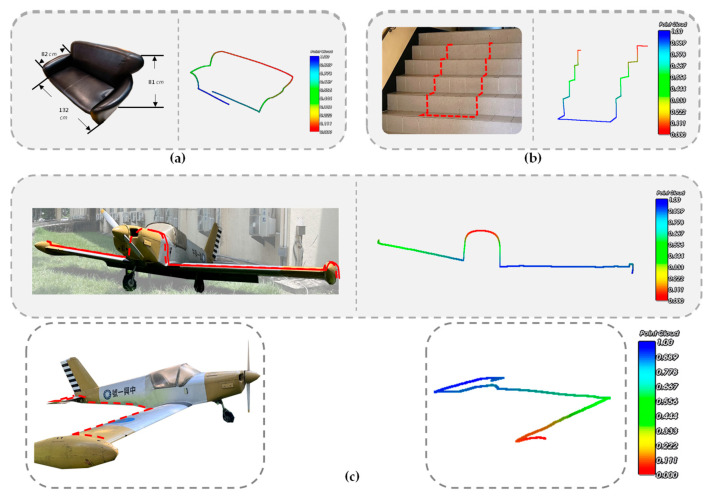
Point cloud acquisition and scanning path generation of large 3D objects (**a**) a sofa, (**b**) stair steps, and (**c**) a light aircraft.

Finally, it was verified that this system can also be applied to the point cloud acquisition and scanning path generation of large 3D objects. A sofa, stair steps, and a light aircraft were selected as measurement targets. Figure 25 shows the corresponding measurement results. It can be seen from the figures that the point cloud trajectory obtained from the scan is consistent with the actual object outline. However, due to the accumulation of measurement errors shown in Equation (12), the measurement data of large objects differs apparently from their actual dimensions, requiring further correction and compensation. Currently, only the 3D appearance can be presented. The contour scanning results shown in Figure 25 are only intended to illustrate the development potential and future scalability of this system. Further efforts are needed to obtain more accurate and reliable quantification measurement results.

## 5. Discussion and Conclusions

This research has successfully developed an optical 3D point cloud acquisition and sketch system with visual display interface based on inertial and displacement sensors.

The system can achieve length measurement with a minimum resolution of 0.01 cm in one dimensional linear movement. In a 2D plane, the system can acquire a complete set of point cloud coordinates. Taking a rectangular contour measurement object of 15 cm × 10 cm as an example, with a total path distance of 50 cm, the actual number of measured point clouds is 3070. The maximum error after analysis is approximately 4.7%; the RMS error is approximately 2.1%. This demonstrates the feasibility of applying the system to planar path measurement. In 3D space, the system can be practically applied to the measurement of 3D objects. Observing the measurement results of the cuboid in Section 4.2, the positions of the outer contour angle deflection of 90° all show a consistent trend. After analysis, the maximum error obtained is approximately 4.8%; the RMS error is approximately 2.3%. This verifies the feasibility of applying the system to 2D and 3D measurement, showing good stability and accuracy.

Another important advantage of this system is that the size of the object being measured is not a limitation. However, due to the cumulative error of the measurement, the measurement data of this system differs apparently from the actual size when scanning larger objects, and can only display the correct outline.

This study has not yet analyzed the impact of different surface features on sensing performance. Therefore, the cumulative error caused by different test surfaces will vary. Consequently, the sensing data differs significantly from the actual object size during long-distance measurements of large objects. The large object contour scanning results shown in this paper are only intended to illustrate the development potential and future scalability of this system. The large object contour scanning results demonstrate, through multiple measurements of stair steps with significant angular changes, that the Mahony algorithm did not experience severe divergence or flipping during continuous long-distance operation, and the system can still maintain the geometric topology of the object. Scanning of light aircraft shows that even under outdoor sunlight, unaffected by complex ambient light, it can still scan the contour of aircraft wings on the order of several meters. In addition, conventional optical 3D scanning equipment is not effective for measuring objects with low reflectivity and black surfaces. The scanning results of a black sofa show that this study still has the advantage of reconstructing the contour changes of the sofa’s shape.

Further accurate quantification results will be an important task and development goal for future research. To make the system more reliable and suitable for industrial applications, some potential improvements are needed, such as sensor calibration, simultaneous localization and mapping (SLAM)-based drift correction, and multi-sensor fusion. In addition, designing a repositioning algorithm based on key feature points (such as identifying object corners, edges, and other features to reset errors), and introducing sparse landmark assisted global optimization might be capable approaches.

## 6. Patents

Invention Patent I764379, Taiwan, China.

## Figures and Tables

**Table 1:** Parameters for the attitude angle calculation.

Parameter	Value (or Action)	Note
sampling rate	~290 Hz	
cutoff frequency of the low-pass filter	20 Hz	
cutoff frequency of the high-pass filter	(0.6, 0.4, 0.4) Hz	*x*, *y*, and *z* axes
PI controller	*K_p_* = 1, *K_i_* = 0.01	
synchronization	use timestamp	accurately calculate the time interval of each piece of data

**Table 2:** Settings of important parameters used in the study.

Sensor	Parameter	Unit	Setting
optical displacement sensor	resolution	cpi	8000
	frame rate	Hz	12,048
	z-height	mm	1.5
	resolution	cm	0.01
accelerometer	full-scale range	g	±2
	resolution	LSB/g	16,384
	noise density	μg/√Hz	400 (@10 Hz)
gyroscope	full-scale range	°/s	±2000
	resolution	LSB/(°/s)	16.4
	noise density	°/s/√Hz	0.005 (@10 Hz)

**Table 3:** Comparison on coefficients of linear regression before and after correction.

Axis	Slope *β*_1_		*R* ^2^	
	B.C. *	A.C. *	B.C. *	A.C. *
+*x*-axis	0.5880	1.082	0.9989	1.000
−*x*-axis	−0.5773	−1.078	0.9992	1.000
+*y*-axis	0.5478	1.105	0.9990	1.000
−*y*-axis	−0.5425	−1.090	0.9989	1.000

* B.C.: before correction; A.C.: after correction.

**Table 4:** Correction results using a displacement of 5 cm.

	Standard Deviation (cm)		Mean Value (cm)		Relative Error (%)		Absolute Error (cm)	
	B.C.	A.C.	B.C.	A.C.	B.C.	A.C.	B.C.	A.C.
*x*-axis	0.108	0.039	9.93	5.612	1.09	0.70	4.93	0.61
*y*-axis	0.172	0.018	10.56	5.73	1.63	0.32	5.56	0.73

**Table 5:** Determination of optimal cutoff frequencies for each axis.

Frequency (Hz)	Relative Amplitude		
	*x*-Axis	*y*-Axis	*z*-Axis
0.0	1.2381	0.0966	0.0597
0.2	0.2532	0.0149	0.0145
0.4	0.2157	0.0232	0.0134
0.6	0.1555	0.0101	0.0064
0.8	0.0988	0.0090	0.0057
1.0	0.0309	0.0091	0.0066
Average Value	0.009	0.005	0.005
Standard Deviation	0.057	0.005	0.004
Threshold Value	0.123	0.015	0.012
Cutoff Frequency (Hz)	0.6	0.4	0.4

**Table 6:** Summary of the measurement results.

Object	Number of Measured Point Clouds	Maximum Error (%)	RMS Error (%)
right triangle	2005	4.4	1.1
rectangle	3070	4.7	2.1
circle	3039	4.1	2.8
cuboid	9685	4.8	2.3
inclined surface	7570	3.5	1.6
curved surface	4402	5.3	2.4

**Table 7:** Comparison of key features of the systems in the literature and the proposed system.

Systems	Sensors	Accuracy	Drift Characteristics	Resolution
D. Grivon [10]	inertial sensors (magnetometer, gyroscope, and accelerometer) optical displacement sensor	2.9% (@ 68 mm)	eliminating long-term drift by using acceleration and magnetic force as reference data through an extended Kalman filter	6.35 × 10^−2^ mm (theoretically @ 400 cpi)
B. Milosevic [11]	inertial sensors (magnetometer, gyroscope and accelerometer) stereo webcam	1% (@ 100 mm)	providing absolute coordinates through the stereo vision system	1.0 mm
T. Stanko [12]	inertial sensors (magnetometer, gyroscope, and accelerometer) incremental wheel	1.15% (@ 1436 mm)	eliminating long-term drift by using acceleration and magnetic force as reference data through a Kalman filter	5.7 mm
This paper	inertial sensors (gyroscope and accelerometer) optical displacement sensor	2.1% (@ 103 mm)	eliminating long-term drift through Mahony algorithm and Butterworth high-pass filter	0.1 mm (after threshold filter) 3.175 × 10^−3^ mm (theoretically @ 8000 cpi)

## Data Availability

The data presented in this article are not currently publicly available but are available from the authors on reasonable request. (e.g., raw data, calibration data, code).

## Abbreviations

The following abbreviations are used in this manuscript:

2D	Two-dimensional
3D	Three-dimensional
MCU	Microprocessor control unit
PI	Proportional–integral
FFT	Fast Fourier transform
SQUAL	Surface quality
SROM	Shadow ROM
CPI	Counts per inch
RMS	Root mean square
SLAM	Simultaneous localization and mapping
